# Immature stages of *Phloeosinus tacubayae* (Curculionidae: Scolytinae): morphology and chaetotaxy of larva and pupa, sexual dimorphism of adults, and developmental time

**DOI:** 10.1093/jisesa/iead104

**Published:** 2023-12-07

**Authors:** Montserrat Cervantes-Espinoza, Enrico Alejandro Ruiz, Gerardo Cuellar-Rodríguez, Ulises Castro-Valderrama, Francisco Armendáriz-Toledano

**Affiliations:** Colección Nacional de Insectos, Departamento de Zoología, Instituto de Biología, Universidad Nacional Autónoma de México, Cto. Zona Deportiva S/N, Ciudad Universitaria, México City, C.P. 04510, Mexico; Laboratorio de Ecología, Departamento de Zoología, Escuela Nacional de Ciencias Biológicas, Instituto Politécnico Nacional, México, Prolongación de Carpio y Plan de Ayala s/n, C.P. 11340, Ciudad de México, México; Laboratorio de Ecología, Departamento de Zoología, Escuela Nacional de Ciencias Biológicas, Instituto Politécnico Nacional, México, Prolongación de Carpio y Plan de Ayala s/n, C.P. 11340, Ciudad de México, México; Departamento de Silvicultura, Facultad de Ciencias Forestales, Universidad Autónoma de Nuevo León, Linares, Nuevo León, México; Departamento de Agricultura y Ganadería, Universidad de Sonora, Km. 21 Carretera Hermosillo-Bahía Kino, 83000 Hermosillo, Sonora, México; Colección Nacional de Insectos, Departamento de Zoología, Instituto de Biología, Universidad Nacional Autónoma de México, Cto. Zona Deportiva S/N, Ciudad Universitaria, México City, C.P. 04510, Mexico

**Keywords:** Scolytinae, morphology, larvae, pupae, bark beetle

## Abstract

The current knowledge of morphology and chaetotaxy of the different developmental stages within the subfamily Scolytinae presents an information deficit that needs to be addressed. Thus, the objective of the present study was to describe, the chaetotaxy and morphology of larvae and pupae, and determine the number of larval instars, the sexual dimorphism in adults, and the development time in *Phloeosinus tacubayae*. The number of larval instars was determined using traditional morphometry of cephalic capsule and multivariate analysis; description of morphology and chaetotaxy of larvae and pupae, and sexual dimorphism in adults was based on light microscopy and scanning electron microscopy photographs; finally, we quantified development time by mean reviews of 10 gallery systems selected randomly in infested logs, in the laboratory. Morphometric analysis of the cephalic capsule allowed the recognition of 3 different instars. Our results showed that the larvae of *P. tacubayae* have unique attributes in the body that differentiate them from other genera of the subfamily for example the epicranial suture is not marked, and differentiated from *Phloeosinus canadensis*, such as a smaller number of setae in the maxillae and without a tergal plate. The pupa had a smaller number of setae on the whole body. The most useful morphological characters to identify a sexual dimorphism in adults were found in the shape and relative position of the seventh and eighth tergites; development time lasted 40 days in total, being the pupal stage the one that took the longest to complete.

## Introduction

Small phytophagous beetles belonging to the subfamily Scolytinae are a diverse group integrated by more than 6,000 spp., which evolved, develop, and live in a wide variety of plant tissues and species, mainly shrubs and trees (Wood 1982, 2007, Kirkendall et al. 2015, Raffa et al. 2015). Their taxonomy is based primarily on adult traits; however, there is a deficit of information related to their immature stages in both larvae as pupa (Hopkins 1909, 1915a, 1915b, Wood 1982, 2007). Description of immature stages of holometabolous insects is paramount in the understanding of different processes, such as xenobiotic detoxification, mortality, survival, feeding specificity, and recognition of taxonomically important characters, among others (BelHabib et al. 2009, Calisto and Morelli 2011, Bozsik and Szőcs 2017, Adesanya et al. 2018, Vendl et al. 2018, Li et al. 2019, Ospina-Garcés et al. 2021). Although it is relatively easy to find immature specimens of bark beetles, they are not usually included in the descriptions, because when several species coexist in the same tissues, it is difficult to ensure kinship among larvae, pupae, and adults. In addition, both larvae and pupae require more careful collection methods and preparation techniques different from those of adults (Hopkins 1909, 1915a, 1915b, Thomas 1957).

There are few studies that integrate morphological and chaetotaxy changes throughout the developmental stages in bark beetles; however, most of them do not present detailed descriptions and comparisons, especially of chaetotaxy. Among the most prominent and notable studies on the Scolytinae in which the immature stages were described are Hopkin’s contributions (1905, 1909), who described the anatomy, general morphology, and terminology of the larval morphological structures, as well as some of the taxonomic attributes recognized in the genus *Dendroctonus* (Erichson, 1836*)(Coleoptera:Scolytinae)*. The study of immature stages in bark beetles has been addressed in 2 ways. First, through the description and comparison of larval morphology in some species and genera (Swaine 1918, Dodge 1938, Chamberlin 1939, Beal and Massey 1945). The most inclusive study was that of Thomas (1957), who compared the morphology of 30 bark beetle species to define attributes for genera and species recognition. The second way was focused on determining the number of larval instars in some bark beetles and analyzing the larval growth in various taxa (e.g. Weber and Mcpherson 1983, Furniss 1995, Masood et al. 2009, Furniss and Kegley 2021). In these studies, the length and width of the cephalic capsule were used to determine the larval instars (Balogun 1970, Logan et al. 1998, Dallara et al. 2012, Bleiker and Régnière 2015). So far, the number of larval instars has been determined in at least 59 species of more than 15 genera (Lekander 1968, Mizell and Nebeker 1979, Weber and Mcpherson 1983, Miller and Borden 1985, Dallara et al. 2012). However, most of them do not consider the estimation of the developmental time of different immature and mature stages or the description of morphology and chaetotaxy together (Balogun 1970, Logan 1998, Bleiker and Regnere 2015). Moreover, the pupa stage is not studied at all, as the detailed description and terminology of this stage are solely based on a study of *Dendroctonus valens* (Leconte, 1860), which in turn has served as an example for the entire subfamily (Hopkins 1905).

Despite the importance of the description of postembryonic development in Scolytinae, it has only been studied in >1% of the species of the group. One of the genera with less information on the morphology of its immature stages is *Phloeosinus* (Chapuis, 1869)*,* whose members feed on scaly conifers of the Cupressaceae family (BelHabib et al. 2007). The general morphology of the *Phloeosinus* larva only has been described and used to distinguish it from other Scolytinae genera (Thomas 1957, Zocchi 1957, Lekander 1968). In addition, the number of larval instars has been estimated in some largest species in the genus, such as *Phloeosinus neotropicus* Schedl, 1939 (= *Phloeosinus serratus* LeConte, 1868) and *Phloeosinus armatus* (Reitter, 1887) (Garraway and Freeman 1981, Mendel 1984), therefore, it is not known if the smallest *Phloeosinus* spp. display the lowest number of instars with respect to larger species. The Mexican cypress bark beetle, *Phloeosinus tacubayae* (Hopkins, 1905) is native to America and widely distributed in Mexico and Central America, as its host *Hesperocyparis lusitanica* (Bartel, 2019) (Cupressales: Cupressaceae) (Cervantes-Espinoza et al. 2022). It is considered an important forest species in natural and urban environments, as well as in plantations because when trees are under water stress, they are more susceptible to colonization by *P. tacubayae* and when their population growth is high, this beetle displays aggressive behavior and produces considerable tree mortality (Fettig 2016). Mexican cypress bark beetle is the smallest species in the genus and develops more than 5 generations per year in Mexico (Cibrián-Tovar et al. 2000). Although *P. tacubayae* is usually one of the most examined beetles in the field of forest parasitology and its life cycle, habitat, importance, and management are generally known (Cibrián-Tovar et al. 2000), the detailed information on its developmental stages is lacking. In this study, we describe for the first time the morphology and chaetotaxy of larvae and pupae of *P. tacubayae*, determine the number of larval stages, explore, and identify new characters that facilitate sex identification; we describe in detail the duration of individual development per instar of this species in laboratory conditions since its previous description is very general.

## Materials and Methods

Specimen collection and identification. Specimens were obtained from 2 Mexican localities in 2 *Hesperocyparis lusitanica* trees: (i) Iztaccíhuatl, Mexico state (19°05ʹ14″N, 98°40ʹ03″W); (ii) Galeana, Nuevo León State (24°49ʹ21″N, 100°05ʹ04″W). In both localities, the trees were felled down and separated into 1-m-long sections. From each site, eleven sections were placed in containers to monitor insect emergence. Logs were kept at room temperature (15–25 °C) and monitored every 2 days to extract specimens. Each time, logs were debarked to expose the wood, galleries, and beetles. To this end, a bark section (10 cm^2^) was removed in both the vertical plane and the entire circumference. Specimens were removed with thin brushes and stored separately in 70% alcohol. To ensure that each stage of development belonged to *P. tacubayae*, the parents found within the gallery system were identified following the keys of Wood (1982). Only the eggs, larvae, and pupae obtained from gallery systems identified as *P. tacubayae* were included. The time required to complete each stage of development was estimated at from weekly revisions for 6 mo of 10 randomly selected gallery systems. To make the observations of immature instars, it was necessary to expose the gallery systems, thus, some larvae and pupae died in each revision event, so the observations were made weekly.

### Number of Larval Instars and Morphology of the Developmental Stages

**Fig. 1.**
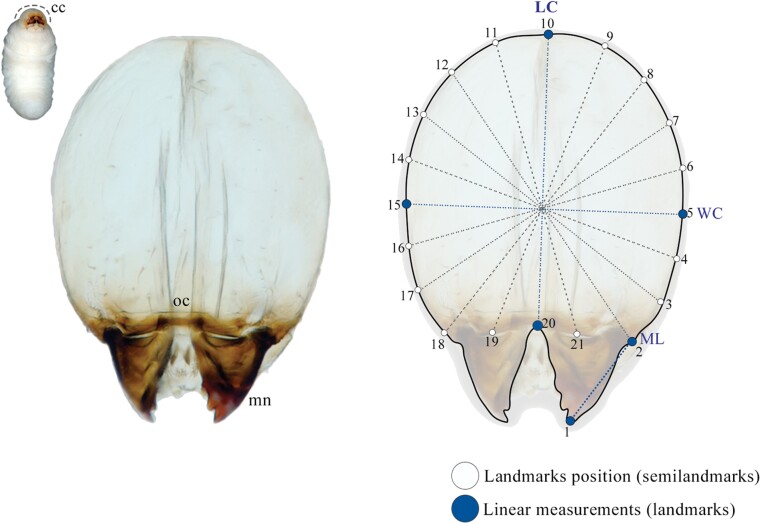
Configuration of landmarks (lm’s) and semilandmarks (sml’s) and fans (dotted lines) used to quantify the morphometric variation of cephalic capsule measurements. **cc**, cephalic capsule; **LC**, length of the cephalic capsule; **ML**, length of the mandible; **mn**, mandibule; **oc**, occipital crest; **WC**, the width of the capsule. Landmarks type 1 (2, 10, and 20), type 2 (1), type 3 (5 and 15), and sml’s (3, 4, 6–9, 11–14, 17–19, and 21).

Specimens of different stages of development were sampled from 7 gallery systems from Iztaccíhuatl and 8 gallery systems from Nuevo León, Mexico. To determine the number of larval instars, a morphometric assay of the cephalic capsule was performed on 120 *P. tacubayae* larvae following the methodology described below. Due to the small size of the cephalic capsules of the larvae of *P. tacubayae*, a slight clarification was performed to observe them in slides mounted with glycerol. Larvae were prepared according to Thomas (1957). Cephalic capsules were separated from the body and mounted in semi-permanent glycerol preparations to facilitate handling. Cephalic capsules were photographed in a frontal position with a phase-contrast microscope at 100× (Carl Zeiss Axioskop with digital camera Axiocam ERC 55). Based on the images of 96 cephalic capsules, morphometric analysis was performed; in each image, 21 homologous points as landmarks (lm’s) and semilandmarks (slm’s) were digitized to define the contour of the cephalic capsule and the length of the mandible, as follows: 3 lm’s type 1 (2, 10, 20), 1 lm’s type 2 (1), 2 lm’s type 3 (5, 15) and 15 sml’s (Fig. 1). Characters from cephalic capsules were chosen since both cephalic width and length are generally used to discriminate larval stages in Scolytinae (Daly 1985). To make the sml’s equidistant and proportional among them (Bookstein 1997, Zelditch 2004, Armendáriz-Toledano et al. 2014), a circle fan with radial lines and equal angular intervals was drawn in each image using the MakeFan6 application version beta (Sheets 2003) (Fig. 1). The landmarks and semilandmarks were digitized into discrete points using the tpsDIG software version 1.40 (Rohlf 2004). Once the landmarks were defined, the centroid size (CS) was extracted (Zelditch 2004), and the following continuous characters were established from form configurations: the length of the cephalic capsule (LC), the width of the capsule (WC), the length of the mandible (ML), and CS of the cephalic capsule (Fig. 1); CS was calculated using Past 1.95 (Hammer et al. 2001).

To evaluate if the variation of the cephalic traits allows the identification of discrete groups corresponding to larval instars, data independence and normality of cephalic traits distribution were tested using the Shapiro–Wilkinson test (Zar 2010), and distribution histograms. To recognize the larval instars, we analyze the multidimensional patterns of morphological variation of cephalic traits by meaning a principal component analysis (PCA), considering each cephalic capsule as an operational taxonomic unit (OTU). PCA was computed from a correlation matrix (Legendre and Legendre 1998), using the 4 characters mentioned above, 3 continuous characters, and the CS. To analyze the individual contribution of characteristics to discriminate among larval instars, the minimum, maximum, mean, and standard error for each continuous attribute were calculated and used for ANOVAs, with respective multiple comparisons (Zar 2010). Finally, when the number of larval instars was established based on the segregation of OTUs in multivariate space, the morphology and chaetotaxy of each instar were described and qualitative characters that could separate the number of instars were sought.

To describe the morphological changes among immature stages (pupa, larva, and egg) and adults, 20 specimens per stage were analyzed and photographed, with an alpha 6000 camera SONY mounted on a stereomicroscope (Stemi DV4 800, Carl Zeiss). Moreover, to describe with greater detail the developmental changes in the external morphology of each stage, and to describe the cephalic capsule chaetotaxy, 5 specimens per stage (larvae, pupa, and adult) were selected for their analysis; the egg was also included but the fixation methods were not effective and were discarded from the SEM analysis; immature stages were prepared in 2.5% glutaraldehyde for 24 h, then washed with buffer twice and gradually dehydrated in 10–100% ethanol, 60 min at each change. Later, the specimens were immersed in 100% ethanol and then placed at the critical point on aluminum foil and gold-plated, for visualization using a scanning electron microscope (SEM, Hitachi S-2469N, Hitachi). The drawings were made freehand from electron microscopy photographs and edited using Corel Draw 2022. The description of the larvae and pupa morphology and the structure of the body at each stage was performed following the nomenclature described by Hopkins (1905) and Thomas (1957); terms and abbreviations for the chaetotaxy used of the mature larva and pupa were based on Scherf (1964), May (1977, 1994), Marvaldi (1998, 1999), and Skuhrovec et al. (2017); for the description of the antenna sensillae of larvae, we used the nomenclature proposed by Pfaffenberger, (1985). The numbers of setae of the bilateral structures also were described for one side.

### Sex Identification and Dimorphic Morphology of the Adult

To identify external characters that allow sex identification in *P. tacubayae*, 100 insects (47 females and 53 males) from both localities (50 per locality) were sexed by genitalia extraction. The external morphology of the head (frons, presence, or absence of keels), pronotum (size and shape), abdomen (number of tergites), and elytral declivity (pubescence: absence or presence; tubercles: size and arrangement) were examined in detail; only the structures that showed a marked and prevalent sexual dimorphism were described and analyzed. Once these characters were identified, 10 specimens of both sexes were examined by scanning electron microscopy (SEM) to improve the observations and detail the morphology of the structures.

## Results

### Morphometric Analysis (Number of Larval Instars)

**Fig. 2.**
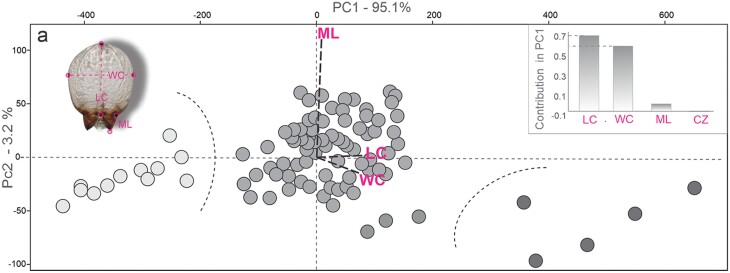
Scatter plot of the principal component analysis (PCA) of 4 continuous characters of the cephalic capsule in *P. tacubayae* larvae. The contributions of each variable are shown on the upper right–hand side. **CZ**, centroid size; **LC**, length of a cephalic capsule; **ML**, length of mandible; **WC**, width of the cephalic capsule.

The PCA of the 4 continuous characters (LC, WC, ML, CZ) of 120 cephalic capsules of larvae recovered 98% of the total variation in the first 2 principal components (PCA1: 95.1%; PCA2: 3.2%; and PCA3: 1.7%) (Fig. 2). The scatter plot between the first 2 PCs showed the segregation of OTUs into 3 nonoverlapping groups according to size of the cephalic capsule, supporting 3 respective larval instars, instar I–III. The attributes that contributed to the most quantity of variation in PC1 were length (LC) and width (WC) of cephalic capsule (Fig. 2). The ANOVAs performed to compare the variation of these characters among 3 instars showed that 3 of the 4 characters present statistically significant differences (LH: *F* = 72.63, df = 95, *P* < 0.001; WC: *F* = 184.9, df = 95, *P* < 0.001; ML: *F* = 28.43, df = 95, *P* < 0.001; CS: *F* = 2.44, df = 95, *P* = 0.145) (Table 1). In all analyses, respective Tukey’s test supported all contrasts (data not shown).

**Table 1.** Mean, standard deviation, and results of ANOVA analyses comparing the morphological measurements of the cephalic capsule of larval stages of *Phloeosinus tacubayae*

Character	Instar I Mean ± SE	Instar II Mean ± SE	Instar III Mean ± SE	*F*	(*P*)
LC	373.73 ± 17.26	645.10 ± 6.01	986.534 ± 42.25	72.63	<0.001
WC	353.40 ± 11.20	576.92 ± 5.73	885.4 ± 38.91	185	<0.001
ML	118.80 ± 6	172.5 ± 3.58	138.93 ± 14.82	28.43	<0.001
CZ	13.18 ± 0.26	12.54 ± 0.08	28.67 ± 16.56	2.442	0.145

Data distribution: LH, cephalic capsule length; WC, cephalic capsule width; LJ, Jaw length; CS, centroid size. *P > 0.05.

### Larval Instars

**Fig. 3.**
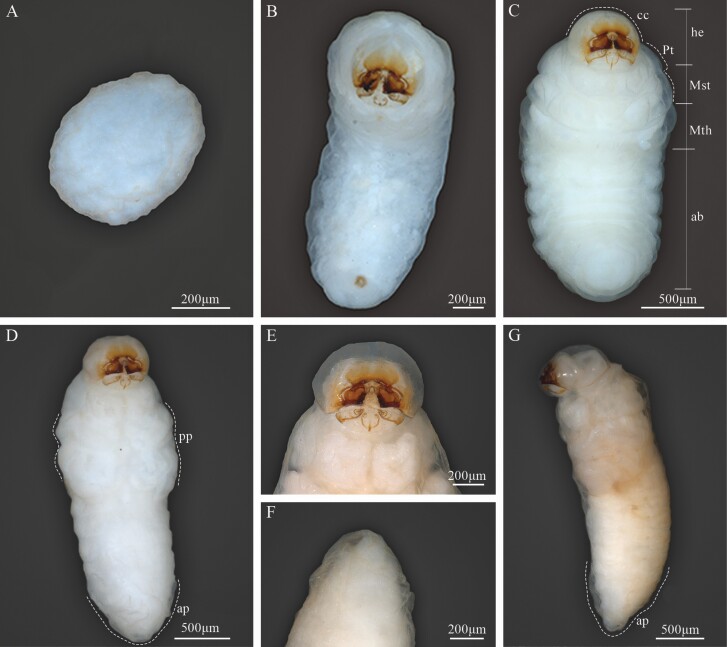
Developmental stages of *Phloeosinus tacubayae*. (A) Egg. (B) Instar larval (InI). (C) Instar larval (InII). (D) Instar larval three (InIII), ventral view. (E) Head. (F) Larva InIII abdomen, apical portion. (G) Lateral view of the prepupa. **ab**, abdomen; **ap**, apical shape; **cc**, cephalic capsule; **he**, head; **pp**, primordia of appendages (legs); **Pt**, prothorax; **Mst**, mesothorax; **Mth**, metathorax.

**Fig. 4.**
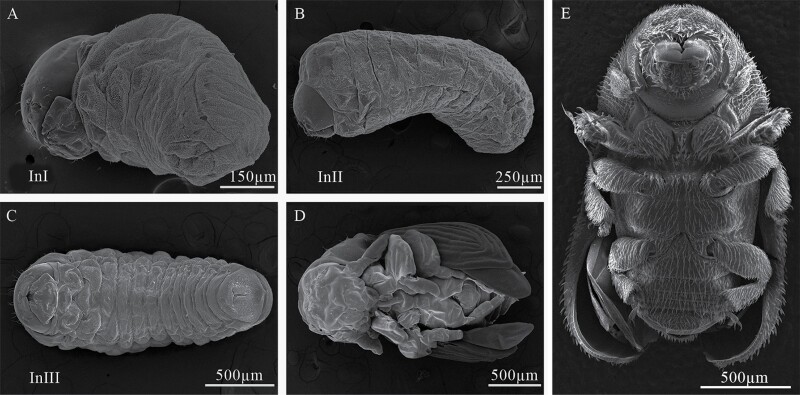
Developmental stages of *Phloeosinus tacubayae*. (A) Instar larval one (InI), lateral view. (B) Instar larval two (InII), lateral view. (C) Instar larval three (InIII), ventral view. (D) Pupa, ventral view. (E) Adult, ventral view.

**Fig. 5.**
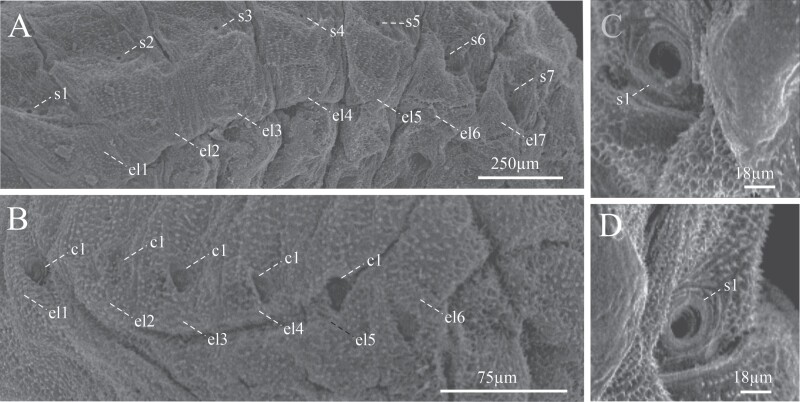
Lateral view of larval instar one. (A) Left lateral view of the segments of the abdomen. (B) Right lateral view of the segments of the abdomen. (C) Frontal view of the left prothoracic spiracle. (D) Prothorax spiracle left frontal view. c, epipleural callus; el, epipleural lobe; s, spiracle.

Larval instar one (InI). White body, with 3 thoracic and 9 abdominal segments (Figs. 3B and 4A); with visible spiracles and epipleural calli (Fig. 5A and B); length and width of cephalic capsule 292–476 µm (LC) and 290–450 µm (WC), respectively, with well-sclerotized and faint black mandibles (Fig. 3B); it is found very close to egg niches.

Larval instar two (InII). Morphology and body color same as InI. The only notable difference is an enlargement of the thoracic segments making this area appear wider; the length and width of cephalic capsules are 539–761 µm (LC) and 467–696 µm (WC), respectively (Figs. 3C and 4B).

Larval instar three (InIII). Body morphology and color like InII (Figs. 3D–G and 4C), length and width of cephalic capsule 896–1,113 µm (LC) and 770–1,001 µm (WC). When metamorphosis begins, the color of the larva changes to light brown and the cephalic capsule begins to contract (Fig. 3E–G), the primordia of the movement appendages are observed, the larva elongates, and the last abdominal segment becomes conical (Fig. 3F and G). A detailed description of the morphology and chaetotaxy of this instar is presented in the following section.

### Morphology of the Developmental Stages and Dimorphic Morphology of Adults

Four developmental stages, eggs, larvae, pupa, and adults of *P. tacubayae* were analyzed and described below (Figs. 3 and 4).

#### Egg.

Eggs are oval, shiny, and white, without ornamentation, 300–520 μm in length (Fig. 3A); throughout their development, they maintain coloration and shape.

### Mature Larva (Stage III)

#### General.

**Fig. 6.**
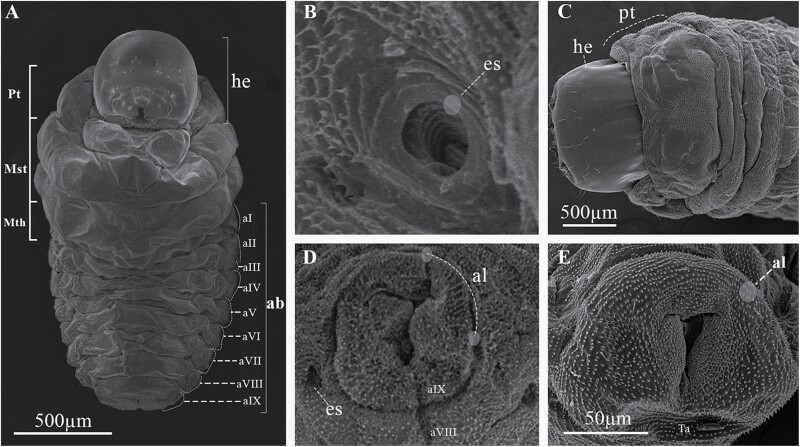
Body characteristics of the larva of *Phloeosinus tacubayae*. (A) Instar III in ventral view. (B) Spiracle of the first thoracic segment. (C) Head and first body segments in dorsal view. (D, E) The frontal ninth abdominal segment in frontal and lateral view, respectively. ab, abdomen; al, anal lobe; es, spiracle; he, head; Mst, mesothorax; Mth metathorax; Pt, prothorax; aI–aIX, abdominal segments.

The body of the larva is wrinkled, cylindrical, legless, ornamented, and divided into 3 segments: head, thorax, and abdomen (Figs. 3D–G, 4C, and 6A), the head is much narrower than the first thoracic segment. All segments have scattered setae, smaller toward the posterior direction (Fig. 6C–E). The thoracic segments are approximately 30% of the length of the abdomen (Figs. 3D and 4C), and the abdomen is composed of 9 well defined segments (Fig. 6A). The last abdominal segment lacks tergal plates and contains only 1 anal lobe (Fig. 6D and E). Each abdominal segment shows 2 single spiracles (Fig. 5C and D), except for those corresponding to the anal lobe (Fig. 6D and E); the spiracles show smooth, ornamental, sclerotized contours with slight brown color, and are larger in the first segments (Figs. 5C, D, 6B, and 9A). In specimens of instar I, each epipleural lobe displays a well defined epipleural callus, which is absent in the later instars (Fig. 5A and B).

#### Head.

**Fig. 7.**
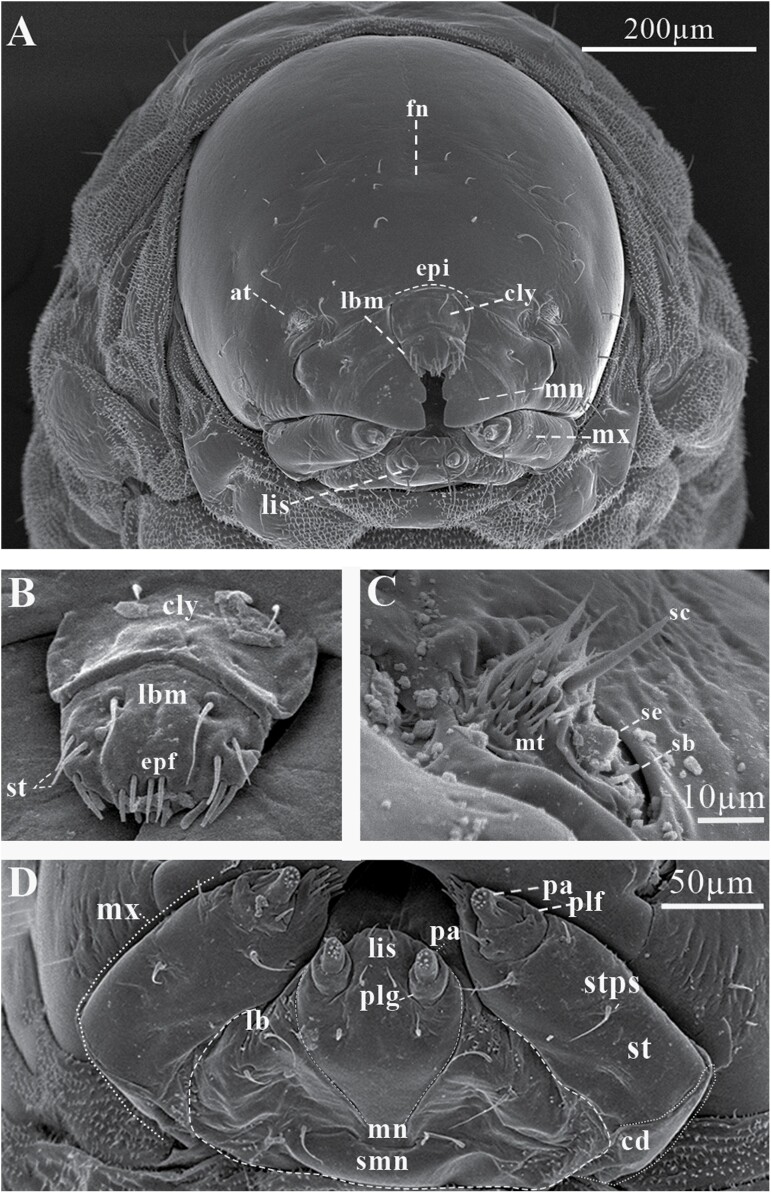
Cephalic capsule of *Phloeosinus tacubayae* larva. (A) Frontal view. (B) Clypeus. (C) Frontal view of the right antenna. (D) Maxilla and ligula. at, antenna; cd, cardo; cly, clypeus; epi, epistoma; epf, epipharynx; fn, frons; lb, labium; lbm, labrum; lis, ligula; mn, mandibula; mx, maxilla; mt, microtrichia; pa, papilla; plf, palpifer; plg, palpiger; plp, palpus; *sb*, sensillum basiconicum; *sc*, sensillum chaetica; *se*, sensorium; smn, submenutum; st, stipe; *stps*, stipital setae.

**Fig. 8.**
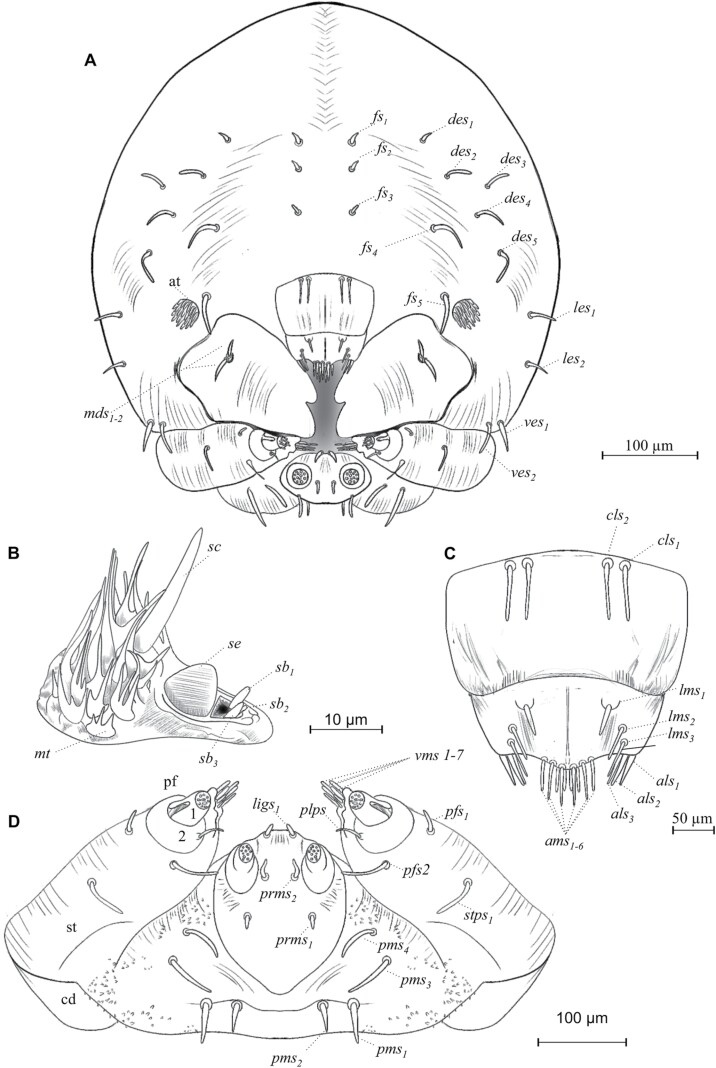
Chaetotaxy of cephalic capsule of mature larva. (A) Frontal view of the capsule. (B) Antenna. (C) Clypeus. (D) Mouth parts. Abbreviations: *als*, anteriolateral setae; *ams*, anteriomedial *se*tae; *at*, antenna; *cd*, cardo; *cls*, clypeal setae; *des*, dorsal epicranial setae; *fs*, frontal epicranial setae; *les*, lateral epicranial setae; *ligs*, ligular setae; *lms*, labral setae; *mds*, mandible dorsal setae; *pf*_*1-2*_, palpifer; *pfs*, palpiferal setae; *plps*, palpar setae; *pms*, palpiferal setae; *prms*, premental setae; *sb*, sensillum basiconicum; *sc*, sensillum chaetica; *se*, sensorium; *st*, stipe; *stps*, stipital setae; *ves*, ventral epicranial setae; *vms*, ventral malar setae; *mt*, microtrichia.

Head sub-oval, with subparallel sides and rounded posterior margins, longer than wide, and partially retracted into the prothoracic dorsum (Fig. 6C), clearly defined by the frons, clypeus, labrum, and a simple epistoma which is the oral margin directly behind the clypeus (Fig. 7A and B). The frons without midcranial suture or poorly defined (Figs.7A and 8A), the frons with 5 frontal epicranial setae (*fs*_*1*_–*fs*_*5*_), distributed in 2 rows, the first one at the center of the frons (*fs*_*1*_–*fs*_*3*_), and 2 more at lateral sides (*fs*_*4*_–*fs*_*5*_); *fs*_*1*_, *fs*_*2*_, and *fs*_*3*_ in a row situated on forehead medially, *fs*_*4*_ situated anteromedially, *fs*_*5*_ situated laterally near the antenna and epistome, *fs*_*1*_–*fs*_*3*_ shorter than *fs*_*4*_ and *fs*_*5*_ (Figs.7A and 8A); lateral epicranial area with 2 setae situated lateral to the cephalic capsule at the level of the mandible (*les*_*1*_–*les2*), and 2 ventral setae (*ves*_*1*_–*ves*_*2*_) on both sides of the head in the inferior area of the epicranium; *les*_*1*–2_ and *ves*_*1–2*_ of the same size and all shorter than *fs*_*5*_ (Fig. 8A); dorsal–lateral area of epicranium with 5 dorsal setae (*des*_*1*_–*des*_*5*_) (Figs. 7A and 8A), the first 2 (*des*_*1*_–*des*_*2*_) are in the upper part of the epicranium, des3 near the middle part of the epicranium, and *des*_*4*_ and *des*_*5*_ are located anteriorly near the antennae; *des*_*1*_ shorter and *des*_*5*_ larger than the others (Fig. 8A).

#### Antennae.

The antenna is located on a groove superior to the mandibles, on each side on the anterior margin of the head, (Fig. 7A), on a membranous convex and hemispherical basal segment (Fig. 7C). On the basal segment, there is a wide conical sensorium (*se*), 3 basiconica sensilla (*sb*), a long chaetica sensilla (*sc*), and at least twelve elongated microtrichia (*mt*) (Figs. 7C and 8B). Sensorium is located among the basiconica sensillum and the cheatica one, measuring 8 µm long and 8 µm wide; the basiconica sensilla, inserted in a pore located below the sensorium, approximately 4 µm length and a 1 µm width, the chaetica sensilla located above the sensorium, approximately 26 µm long and 2µm wide, surrounded by microtrichia; each microtrichium display 3 long prolongations approximately from 8 to 12 µm long and 1 µm wide, the base of each microtrichium 4 µm wide (Figs. 7C and 8B).

#### Clypeus.

Trapezoidal in shape, twice as wide as long, and tapers towards the apical margin, which is emarginate, its connection with the epistoma is continuous, lateral sides are rounded to an acute impressed basal angle at the mandibular condyle (Fig. 7B), with 2 pairs of long clypeal setae, similar in length (*cls*_*1*_–*cls*_*2*_) at each end near the base (Fig. 8A and C).

#### Labrum.

Labrum prominent, wider than long, slightly narrower but longer than clypeus (Fig. 7B), with an anterior margin bisinuate and rounded apical margin; with 2 lines anteroposterior of 3 setae each (*lms*_*1–3*_), *lms*_*1*_ situated close to clypeus margin, *lms*_*2*_ situated anteromedially, and *lms*_*3*_ situated anterolaterally; *lms*_*1*_ longer than *lms*_*2*_ and *lms*_*3*_ (Fig. 8A and C).

#### Epipharynx.

Located in the ventral-apical area of the labrum; with 3 anterolateral setae (*als*_*1–3*_), unequal in length, *als*_*1–2*_ shorter than *als*_*3*_. the ventral part of the epipharynx could not be visualized so the setae in this area are not described in detail; in the dorsal view of the labrum, almost 10 anteromedial setae can be recognized (*ams*_*1–10*_) (Figs. 7B, 8A, and C).

#### Mandibles.

Symmetrical, broad, apically tridentate (Fig. 7A), dorsally with 2 mandible dorsal setae (*mds*_*1–2*_); both setae of medium size like *des*_*2*_ (Figs. 7A and 8A).

#### Maxillae.

Stipe attached to the cardo, which is a semicontinuous piece divided distally into a laciniate lobe and maxillary palpi (not divided into stipe and subgalea) (Figs. 7A and D); the laciniate lobe is armed with 7 small soft ventral malar papillae (*vms*_*1–7*_), ‘papilla’ *sensu*de la Torre-Bueno et al. (1989) or lacinal teth *sensu* (Hopkins 1909) (Figs. 7D and 8D), the dorsal malar setae (*dms*) are not visible; in each stipe at the middle part occurs 1 stipital setae (*stps*_*1*_) and in the distal part next to the palpifer, 2 more palpiferal setae *pfs*_*1–2*_ are present; *stps*_*1*_ and *pfs*_*2*_ very long 3 times longer than *pfs*_*1*_; maxillary palpi integrate by 2 palpomeres as single telescopic joint, the first one with a palpomere seta (*plps*) and the second with apical papillae (Figs. 7D and 8D).

#### Labium.

Attached to a submental lobe (Fig. 7D); the latter has 2 pairs of setae per side, 1 of them anterior (*pms*_*3*_–*pms*_*4*_), and the other posterior one (*pms*_*1*_–*pms*_*2*_), anterior seta larger than posterior (Fig. 8D). The labium is divided into mentum, ligule, and a pair of palpi; the mentum is a median triangular plate, and its anterior and posterior sections are produced, the anterior one is between the palpi and supports the ligule, the posterior section is rounded by the submental lobe (Fig. 7D); on the anterior and middle sections of mentum occur 2 pair of setae, *prms*_*1*_, and *prms*_*2*_, respectively, mentum seta are shorter than those from submental lobes (*pms*_*1–4*_) (Fig. 8D); ligule with 1 pair of apical setae (*ligs*_*1*_) (Fig. 8D). Palpi located at the anterior angles of the mentum, divided into palpiger and labial palpus, the last one with 2 palpomeres, the distal one with until 9 papillae (Figs. 7D and 8D).

#### Thorax.

**Fig. 9.**
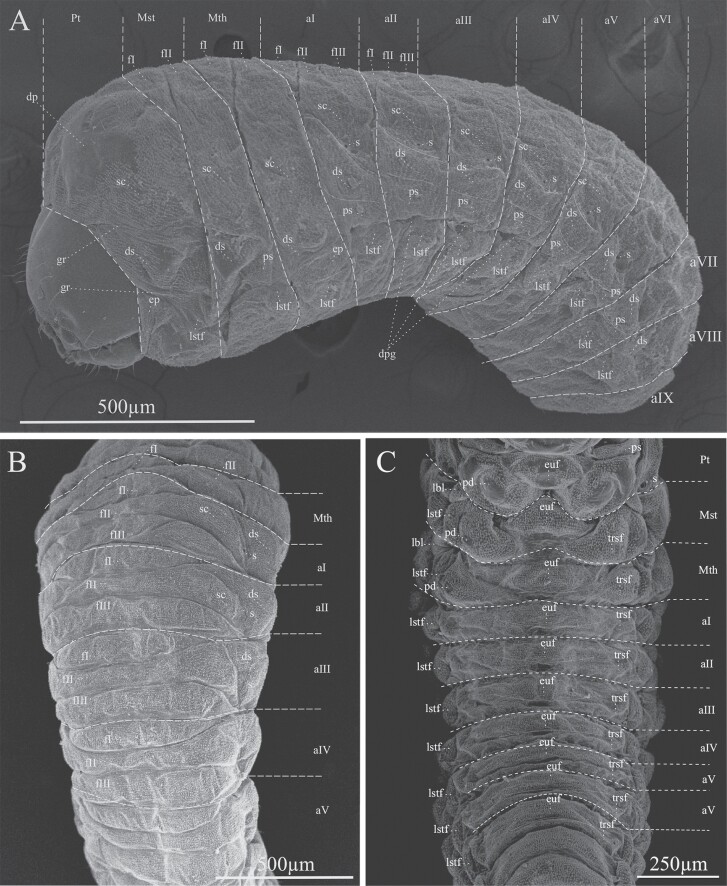
Thorax morphology of *Phloeosinus tacubayae* larva. (A) Lateral view. (B) Dorsal view. (C) Ventral view. Abbreviations: aI–aIX, abdominal segment; dp, dorsal plate; dpg, transversal anterior groove; ds, dorsopleural lobe; ep, epipleura; euf, eusternal fold; fI–fIII, folds; gr, ventral grooves; lbl, lobule; lstf, latero-sternal fold; Mst, mesothorax; Mth, metathorax; pd, area pedal; ps, pleura; Pt, prothorax; s, spiracle; sc, scutellar lobe; trsf, transversal fold.

**Fig. 10.**
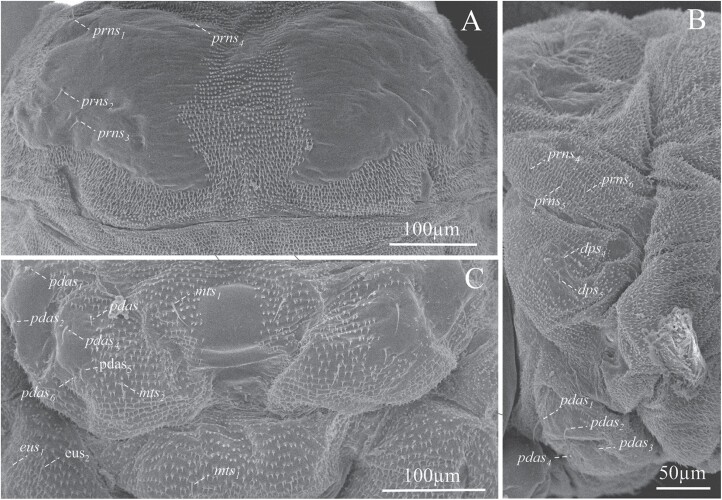
Prothorax. (A) Dorsal view. (B) Lateral view. (C) Ventral view of prothorax and mesothorax. ***pdas***, pedal setae; ***mts***, mesosternal setae; ***eus***, eusternal setae; ***dps***, dorsopleural setae; ***prns***, pronotal setae.

Prothorax undivided, larger than meso- and metathorax (Fig. 9). Dorsum conformed by a scutellar lobe (sc), and a smooth dorsal plate at the center (dp) (Fig. 9A); meso- and metathorax are distinctly longer than abdominal segments I–III (Fig. 9), both divided transversely into 2 folds fI and fII, or prescutal and scutellar lobes *sensu*Hopkins (1909), respectively, the first one (fI) on the anterior region, elliptical in shape, delimited by a median groove, and the second one (fII) on the posterior region, extending the full width of the segment (Fig. 9A and B). Thoracic pleuron integrated by 2 folds, dorso pleural (ds) and pleura (ps), or scutellar and epipleural (ep), respectively, sensu Hopkins (1909); dorsopleural fold delimited dorsally and ventrally by grooves (gr) (Fig. 9A); thoracic segments are not divided from sternum by a deep grove such as the abdominal segments (Fig. 9A). Sternal area of thoracic segments are divided into 4 folds delimited by deep groves (Fig. 9C); anteriorly with a triangular eusternal fold (euf), with rounded vertices; on the posterior end, a transverse fold (trsf), narrower in the middle, in area pedal (pd) with a smooth area rounded on each side, or foot calli; on either said of the median a latero-sternal fold (lstf) occurs; 1 small rounded lobe (lbl) is present, on the anterior edge of latero-sternal folds; the first 3 lobes (eus, trsf, and lstf) were recognized by Hopkins (1909) as sternal, sternellar, hypopleural, respectivelly. Spiracles are unicameral (Fig. 5C and D); the cuticle is densely spiculated and with distinct thorn-like cuticular processes, primarily on dorsal parts but also on pleural parts (Figs. 9 and 10).

**Fig. 11.**
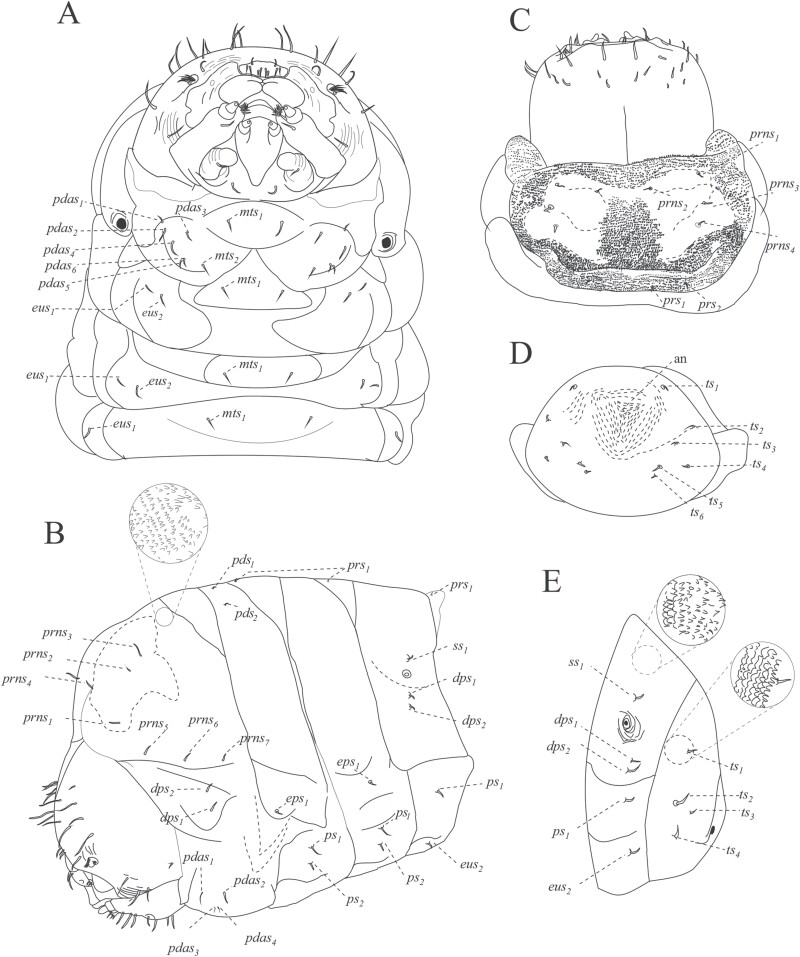
Chaetotaxy of thorax and abdomen of mature larva of *Phloeosinus tacubayae*. (A) Thorax, ventral view. (B) Thorax and abdomen, lateral view. (C) First thorax segment, dorsal. (D) Last abdominal segment, posterior view. (E) The last 2 abdominal segments, lateral view. Abbreviations: an, anus; *dps*, dorsopleural setae; *eps*, epipleural setae; *eus*, eusternal setae; *mts*, mesosternal setae; *pdas*, pedal setae; *pds*, postdorsal setae; *prs*, prodorsal setae, *prns*, pronotal setae; *ps*, pleural setae; *ss*, spiracular setae; *ts*, sternal setae.

Prothorax with 4 pronotal or prodorsal setae (*prns*_*1*_–*prns*_*4*_) on the smooth area of scutellar lobe (Figs. 10A and 11C): *prns*_*1*_ near lateral–anterior margin, slightly smaller than *prn*_*2*_; *prn*_*3*_ and *prns*_*4*_ aligned near posterior margin; 3 more setae occur on lateral sides of the scutellar lobe (*prns*_*5*_, *prns*_*6*_, and *prns*_*7*_);2 dorsopleural setae (*dps*_*1*_–*dps*_*2*_) located on the upper area of spiracles (Figs. 10B and 11B); on the dorsum of the mesothorax in fold I 1 prodorsal setae (*prs*) and in foldII 2 postdorsal setae (*pds*_*1-2*_); pleura of mesothorax and metathorax with 3 setae, 1 of them in epipleural lobe (*eps*_*1*_) and the other 2 in pleural one (*ps*_*1*_–*ps*_*2*_) (Fig. 11B). In the prothorax, a sternal area with 8 setae (Figs. 10B, C and 11A, B): 1 on mesosternal fold (*mts*_*1*_), 6 pedal setae on foot calli (*pdas*_*1*_ –*pdas*_*6*_), and 1 more (*mts*_*2*_) on the posterior section of the transverse fold, where the cuticle is densely ornamented with thorn-like cuticular processes (Figs. 10C and 11A); mesothorax and metathorax with a setae on mesosternal fold (*mts*_*1*_) and 2 more on eusternal setae (*eus*_*1*_–*eus*_*2*_) (Figs. 10C and 11A).

#### Abdomen.

With 9 segments (Fig. 9A); abdominal segments I–IV are almost equal length, and subsequent abdominal segments decrease gradually to the terminal parts of the body. With 8 pairs of lateral unicameral spiracles, those from I–VII segments are similar, obliquely caudal, spiracles of segment VIII smaller (Fig. 9A). The dorsum of the first 8 abdominal segments is divided into 3 folds (fI–fIII), the first one, on the anterior region, elliptical in shape, delimited posteriorly by a complete transversal anterior groove (dpg), the second one on the middle region, delimited posteriorly by a transverse incomplete median grove, not extending the full width of the segment (Fig. 9A and B). The delimitation of the folds on segments VIII and IX is discrete, and the tenth segment is represented by the anal lobe (Fig. 6D and E); segments I to VIII with dorsopleural (ds) and pleural (ep) lobes, indistinctly delimited by grooves proceeding anteriorly (Fig. 9A); sternal area of abdominal segments is divided into tree folds delimited by deep groves (Fig. 9C); anteriorly with an oval medio sternal fold (euf), on the posterior end a transverse fold (trsf), narrower in the middle and on either said of the median fold, a latero-sternal fold (lstf) occurs.

Cuticle is also densely spiculated and with distinct thorn-like cuticular processes (Fig. 9). Abdominal segments I–IX with chaetotaxy similar, dorsally with 1 prodorsal setae (*prs*_*1*_) in each segment; laterally with 1 short spiracular setae (*ss*), 2 setae (*dps*_*1*_–*dps*_*2*_) in dorsopleural lobe, *dps*_*2*_ immediately below *dps1* (Fig. 11B), in latero-sternal lobe, 1 seta is present (*ps*_*1*_) (Fig. 11B); ventrally with 1 setae on mesosternal fold (*mts*_*1*_) and 1 eusternal setae (*eus*_*1*_) (Fig 11A); epipleural callus not evident, only distinguishable for being an area with denser cuticle, in the first larval instar is more noticeable. Abdominal segment IX in frontal view with 6 short sternal setae (*ts*_*1–6*_) 1 in the superior part (*ts*_*1*_), 1 in the part media (*ts*_*2*_), 3 in the part inferior on each anal lobe (*ts*_*3*_*–ts*_*5*_), and 1 very short seta (*ts*_*6*_) on the dorsal anal lobe (Fig. 11D and E).

### Pupa

**Fig. 12.**
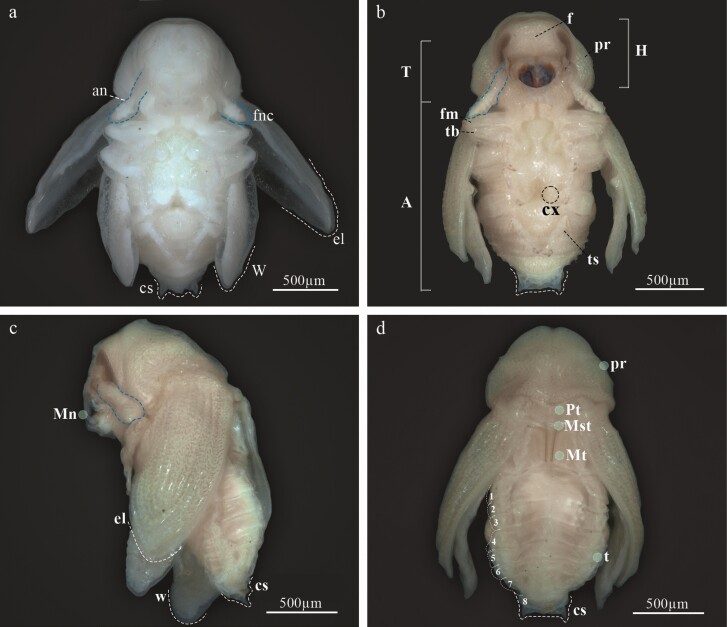
Pupa of *Phloeosinus tacubayae*. (A) Ventral view. (B) Pupa turning yellow as it matures. (C) Lateral view. (D) Dorsal view. **1–8**, tergites; **a**, abdomen; **an**, antenna; **cs**, caudal spinae; **cx**, coxae; **el**, eliter; **f**, frons; **fm**, femur; **fnc**, funiculus; **h**, head; **mn**, mandible; **mst**, mesothorax; **mt**, metathorax; **pr**, pronotum; **Pt**, prothorax; **T**, thorax; **tb**, tibia; **ter**, tergites; **ts**, tarsus; **w**, wing.

**Fig. 13.**
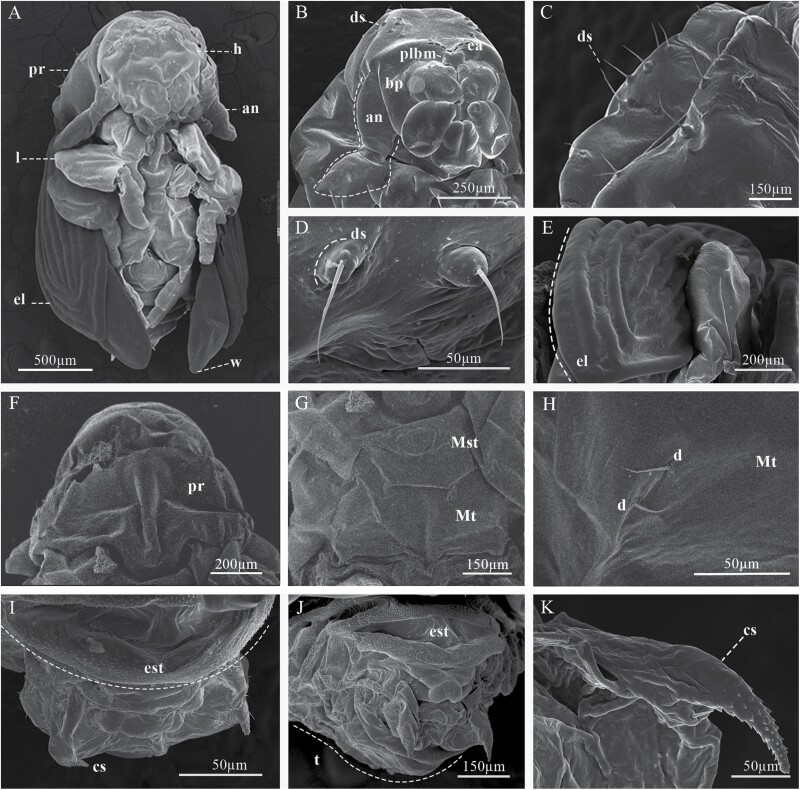
Morphology of the pupa of *Phloeosinus tacubayae*. (A) Ventral view. (B) Head with setiferous tubercles in the frontal and lateral area. (C) Head, setiferous tubercles. (D) Setiferous tubercles. (E) Eliter. (F) Pronotum, dorsal view. (G) Mesothorax and metathorax, dorsal view. (H) Metathorax, dorsal view. (I) Last pair of sternites, posterior view. (J) Last abdominal segment, apical view. (K) Caudal spine. **an**, antenna; **bp**, mouth buds; **cs**, caudal spine; ***d***, dorsal setae; ***ds***, discal setae; **ea**, epistomal area; **el**, eliter; **est**, sternites; **fnc**, funiculus; **he**, head; **l**, leg; **Mst**, mesothorax; **Mt**, metathorax; **plbm**, pseudolabrum; **pr**, pronotum; **t**, tergites; **w**, hing wing.

At the beginning of metamorphosis, the pupa is white, without apparent sclerosis, appendages are present, as well as antennae, wings, and legs, the last with femur, tibia, tarsus, and caudal spines (Figs. 4D and 12A, C). At the end of metamorphosis, cream body color, evident sclerotization, tagmata well defined, the eyes, mandibles, and tarsal claw with sclerotic black color, segmentation of antennae evident, conspicuous elytral sculpture, caudal spines reduced and the last abdominal tergite is already visible (Figs. 12B–D and 13A).

#### Head.

**Fig. 14.**
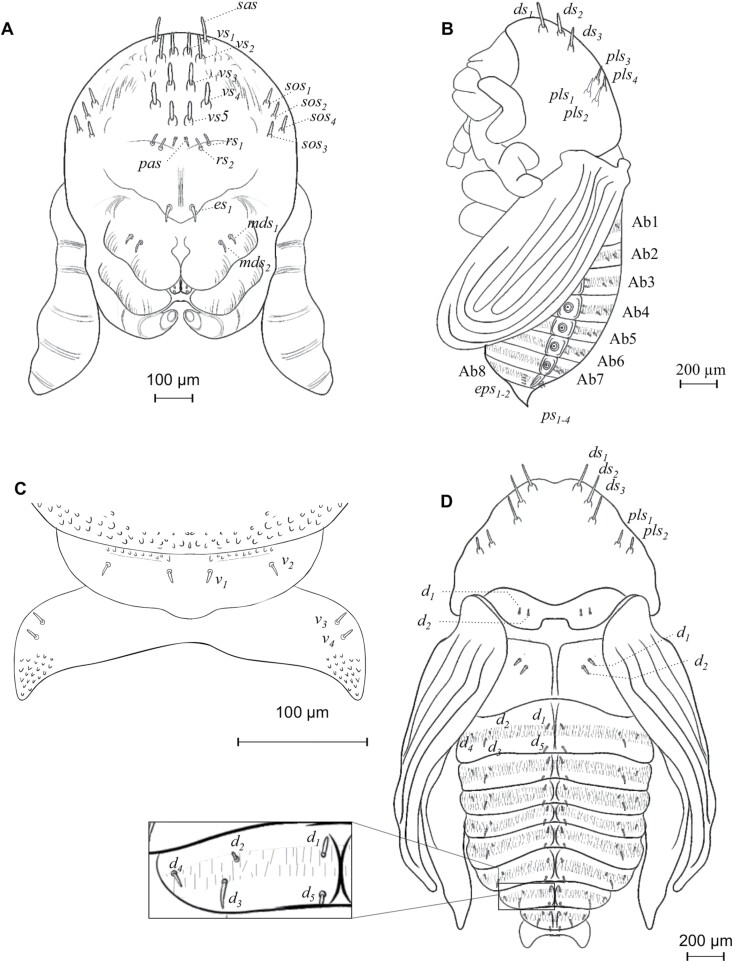
Morphology and chaetotaxy of the pupa of *Phloeosinus tacubayae*. (A) Head, ventral view. (B) Lateral view of pupa. (C) Last abdominal segment and caudal spines, ventral view. (D) Pupa, dorsal view. **Ab**, abdomen; ***d***, dorsal setae; ***ds***, discal setae; ***eps***, epipleural setae; ***es***, epistomal setae; ***mds***, mandibular setae; ***pas***, postantennal setae; ***pls*** posterolateral setae; ***ps***, pleural setae; ***rs***, rostral setae; ***sas***, super apical setae; ***sos***, super orbital; ***v***, ventral setae; ***vs***, vertical setae.

Almost completely covered by the pronotum in dorsal view, short rostrum; the antennae, mandibles, maxillae, and labium are well defined within pads, the eye visible at the end of metamorphosis (Fig. 13B and C). An epistomal bristle-pad or pseudolabrum *sensu*Hopkins (1909) is present, at the upper area of mandibles; the antennae extend to the second pair of legs, and the funiculus extends beyond the lateral margins of pronotum (Fig. 13A and B). On each side of the vertex with dorsal setae, on the upper frons area, 1 anterior setae (*sas*), 5 setae occur in an anterior–posterior direction (*vs*_*1*_–*vs*_*5*_) (Fig. 14A); they are the largest setae on the head, in the median region of the frons one pair of short postantennal setae (*pas*), they are the shorter setae on the head, and 2 rostral setae are present (*rs*_*1*_–*rs*_*2*_), the lateral areas of the head with 4 super orbital setae (*sos*_*1*_–*sos*_*4*_) (Figs. 13B and 14A); epistomal bristle-pad with 1 epistomal setae (*es*_*1*_) (Fig. 14A); mandibula with 2 setae (*mds*_*1–2*_) (Fig. 14A); setae on the head straight or slightly curved, and shorter than setae on the pronotum.

Prothorax moderately spiculated, with discal setae tubercles, the setae are more prominent than those of the head or body and are much more noticeable than in the larval stages (Figs. 12D and 13C–D). Pronotum with 2 anteroposterior lines of setae distributed on the dorsal area; 3 discal setae (*ds*_*1*_–*ds*_*3*_) anteriorly (Fig. 14C), 4 posterolateral setae (*pls*_*1*_–*pls*_*4*_), equal in length, forming a group medially, located along the posterior margin of pronotum (Figs. 14B–C). Mesothorax is sub-rectangular with a rounded scutellum and a median process of the scutellum well defined; 2 dorsal setae (*d*_*1*_–*d*_*2*_) occur at lateral sides (Fig. 14C); the mesothoracic spiracle located between the posterior lateral margin of the prothorax and the anterior ventral angle of the elytral pad. Metathorax is prominent and well-differentiated, not so high in its middle part, with a sulcate area at the middle and 1 pair of posterior tergal setae on each side (*d*_*1*_–*d*_*2*_) (Figs. 13F–H and 14C).

#### Abdomen.

Tergites are well defined (Figs. 12B, D, 14B, and C), the 8 tergites visible dorsally, setae on abdominal segments whit thorn-like cuticular processes; segments I and II slightly wider than others; in each tergite, a horizontal median line of cuticular processes occurs, in each line, 5 dorsal setae (*d*_*1*_–*d*_*5*_) forming 1 transverse row close to posterior margin are present, *d*_*3*_ bigger than the others (Fig. 14C); in lateral view 2 epipleural setae (*eps*); in 8 tergite a group of 4 pleural setae (*ps*_*1–4*_); 7 annular spiracles visible on each side of the body, in segments eighth and ninth these elements cannot be distinguished (Fig. 14B); last sternite with 4 ventral setae (*v*_*1*_–*v*_*4*_) all the similar size (Figs. 13I–K and 14D); urogomphi in tergite 9 has caudal spines, with the serrate surface, the base of each caudal spine with ventral scales and 1 seta (Figs. 13J and K).

### Morphology and Description of Sexual Dimorphism of Adults

**Fig. 15.**
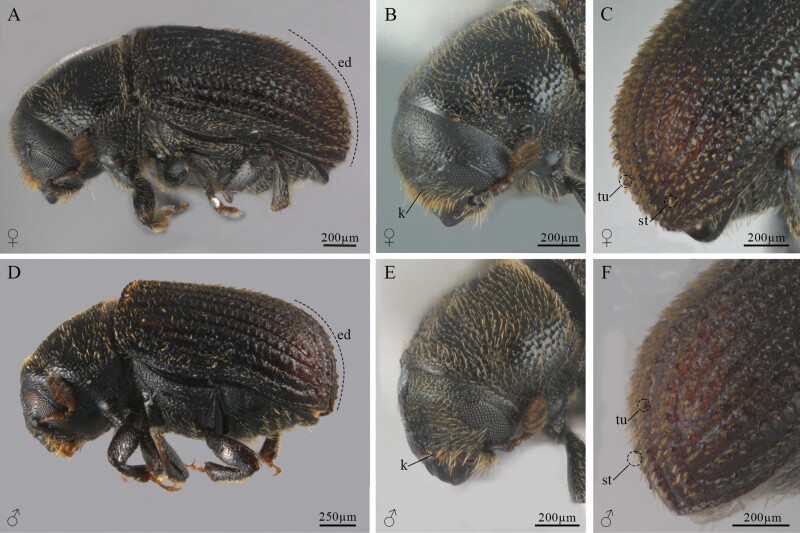
Female and male adults of *Phloeosinus tacubayae*. (A) Female, lateral view. (B) Female head. (C) The elytral declivity of female. (D) Male in lateral view. (E) Male head. (F) The elytral declivity of male. **ed**, elytral declivity; **k**, keel; **st**, setae; **tu**, tubercle.

**Fig. 16.**
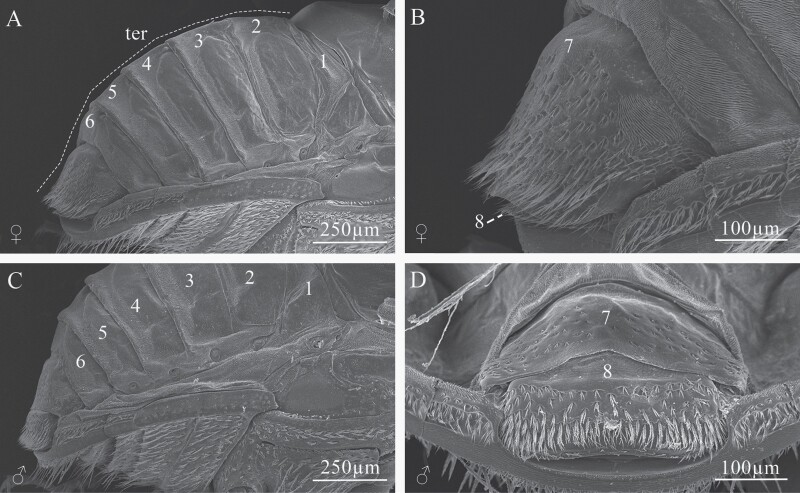
The abdomen of *Phloeosinus tacubayae* (female and male). (A) Female in lateral view. (B) Close-up of the last tergites in female. (C) Male in lateral view. (D) Close-up of the last tergites in male. **ter**, tergites; **1–8**, number of tergites.

Sexually dimorphic characters described in the literature are the midline of the frons or ellipsoidal declivity less pubescent and shiny in males (Hopkins 1905); and for females: planoconvex frons, interstrial tubercles on the disc higher than those of males, longer declivity scales, each about 3–4 times longer than wide (Wood 1982). However, these attributes are highly variable, and it is not possible to discriminate the sexes using them (Fig. 15); the only characteristics that were maintained to recognize the sexes were the size and shape of the seventh and eighth tergites in all beetles analyzed (Fig. 16). The seventh tergite in females is oval, longer than wide, and larger than the eighth tergite, which is completely covered by the seventh tergite (Fig. 16A and B); whereas in males, the seventh tergite is short, wider than long, the eighth tergite is short, longer than wide and seals with the last sternite; this segment is not covered by the seventh tergite as in females (Fig. 16C and D).

### Development Time

**Fig. 17.**
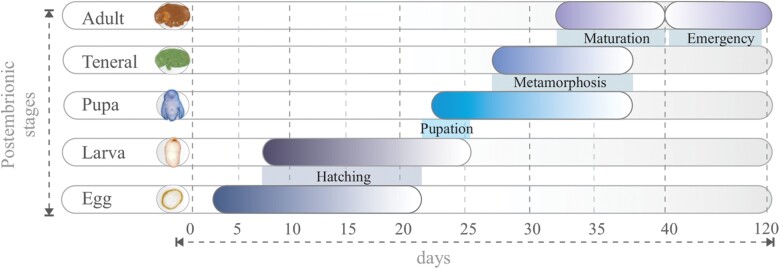
Life history and development time of *Phloeosinus tacubayae* under laboratory conditions.

From the weekly review of the gallery systems, we quantified the time required for the complete development of the insect (from the egg to the adult stage); that is, depending on the development stage we recorded the duration of each stage; the development time for the entire life cycle was 40 days (Fig. 17). Egg deposition was recorded from day 2 to 21, (19 days) and hatching from day 8 to day 21 (15 days). The period in which the 3 larval stages can be found was from day 8 to 26, (18 days); larvae began metamorphosis from day 23 to 26, (3 days). Pupae were present from day 23 to 37, (14 days), and became images from day 27 to 37, (ten days). Teneral were present from 27 to 37 days (ten days), and in 5 days were mature. Adults were present within pupal chambers from day 33 until day 40, 7 days in total. Emergence was recorded from day 43 to 121, 72 days in total. Each of these stages overlaps with each other and there may be different stages of the life cycle in the same gallery.

## Discussion

### Larval Instars

Determination of larval stages in bark beetles is an important aspect for describing the age distribution of populations, assessing the climatic suitability of new habitats, predicting potential dispersal, quantifying stage-dependent mortality, calibrating phenological and population models, as well as for integrated pest management (Régnière et al. 2012, Bleiker and Régnière 2015). Larval instars of bark beetles are determined by measuring the linear features of the cephalic capsule using a growth factor (FC) (Dyar 1890, Balogun 1970, Sprick and Gosik 2014, Gosik et al. 2019a). Cutoff points for each instar have been recognized by histograms (Bleiker and Régnière 2015), nonlinear least-squares regressions (Logan et al. 1998), and by determining free distribution models (Dallara et al. 2012). However, these methods have been criticized for requiring large numbers of specimens and selecting appropriate frequency classes. Our study constitutes the first multivariate approach to estimate the instar number in bark beetles. Photographing the cephalic capsules allowed us to recognize measurement errors, as well as to standardize cutoff points (i.e. nonoverlapping groups in multivariate space). Through multivariate analysis, measuring 4 morphometric traits, 3 groups were recognized which are equivalent to 3 larval instars. This conforms to the number of larval instars in Scolytinae which can be from 2 to 5 (Dallara 2012); among *Phloeosinus* members, *P. tacubayae* display represents the lowest larval instar number, compared with *P. armatus* (Mendel 1984), that develops and displaying 4 larval instars. *Phloeosinus tacubayae* is the smallest species of the genus, so our results agree with trends observed in other Coleoptera, whose smaller species have fewer larval instars (Gosik et al. 2020, Skuhrovec et al. 2022).

### Morphology and Chaetotaxy of Larvae

Larval morphology has been extensively studied in the family Curculionidae and is crucial for establishing taxonomic characteristics and supporting its higher classification (Marvaldi et al. 2002, Skuhrovec et al. 2017, Gosik et al. 2019a, 2020). Compared to other members of Curculionidae, the morphology of pupae and larvae in Scolytinae have been poorly documented and described in a few taxa (Hopkins 1909, Thomas 1957). The most detailed description of larvae and pupae of Scolytinae corresponds to *Dendroctonus valens* (Hopkins 1909*),* and the most extensive comparison of larval characters (number and arrangement of setae, coloration of setae on the labrum, epipharynx, mandible, maxilla, labium, thorax, and abdomen) was performed in 30 species of bark beetles, representing 15 genera (*Crypturgus* (Erichson, 1836*), Polygraphus* (Erichson, 1836) (Coleoptera:Scolytinae)*, Dendroctonus, Trypodendron* (Stephens, 1830) (Coleoptera:Scolytinae)*, Gnathotrichus* (Eichhoff, 1869) (Coleoptera:Scolytinae)*, Conophthorus* (Hopkins, 1915) (Coleoptera:Scolytinae)*, Phloeosinus, Hylurgops* (LeConte, 1876) (Coleoptera:Scolytinae)*, Pityophthorus* (Eichhoff, 1864) (Coleoptera:Scolytinae)*, Orthomicus* (Ferrari, 1867) (Coleoptera:Scolytinae)*, Dryocoetes* (Eichhoff, 1864) (Coleoptera:Scolytinae)*, Ips* (De Geer, 1775) (Coleoptera:Scolytinae)*, Pityogenes* (Bedel, 1888) (Coleoptera:Scolytinae)*, Scolytus* (Geoffroy, 1762) (Coleoptera:Scolytinae)*, Pityokteines* (Fuchs, 1911) (Coleoptera:Scolytinae)), including *Phloeosinus canadensis* Swaine, 1917 (Thomas 1957). In *P. tacubayae*, some larval attributes were recognized in common with these Scolytinae members, such as apodous, curved body shape, hypognathous head, thoracic and abdominal segments composed of integumental folds (not separated by distinct sutures), thoracic segments on the ventral surface with lobes having a sclerotized area with setae, and the surface of the integument covered with fine, backward projecting, cuticular spines. The last 2 attributes are important as adaptations in Scolytinae because they are associated with the locomotion within larval tunnels (Hopkins 1909, Thomas 1957).

Some characteristics of larval morphology and chaetotaxy also were common between *P. tacubayae* and *P. canadensis*, and different with respect to other bark beetles (see Table 2). Most Scolytinae have head-free capsules, but *P. tacubayae* and *P. canadensis* display head capsules partially retracted into the prothorax. Also, these species present the dorsal epicranial setae located more anteriorly than in other Scolytinae and both display cephalic capsules slightly longer than broad, with subparallel sides and rounded posterior margins; meanwhile, most Scolytinae have curving sides and broadly rounded posterior margins of the cephalic capsule (Thomas 1957). In both *P. tacubayae* and *P. canadensis* the most prominent larval attributes to differentiate them respect other Scolytinae are the unmarked epicranial sutures, less pigmented head capsule, the less ornamented body cuticle, the less excluded spiracles, and the absence of tergal plate, in comparison with other taxa, such as *Dendroctonus* (Thomas 1957, Víctor and Zúñiga 2016)*, Ips* (Balogun 1970, Miller 1985), *Scolytus* (Kaston 1937), *Drycoetes*, and *Pityophthorus* (Thomas 1957). The pattern of less ornamented cuticles in *Phloeosinus*, also is reflected in less abundant or absent chaetotaxy on different body parts, such as posterior epicranial seta which is absent in *P. tacubayae*, and present in other Scolytinae members, including *P. canadensis* (Thomas 1957), a smaller number of setae on the pedal lobe on metathorax, in *P. canadensis* there are 3 setae and in *P. tacubayae* are absent (see Table 2). When compared with other members of the family Curculionidae the tendency to reduce the number of sensilla and cuticular ornamentations in the 2 *Phloeosinus* spp. is more marked, for example *Bagous claudicans* (Boheman, 1845) (Coleoptera: Curculionidae), *Orobitis cyanea* (Linnaeus, 1758) (Coleoptera: Curculionidae), *Sciaphilus asperatus* (Bonsdorff, 1785) (Coleoptera: Curculionidae) present a more ornamented cuticle or marked epicranial sutures (see Table 2) (Gosik et al. 2019a, Gosik and Sprick 2022).

**Table 2.** Larval setal index of the genera and species with which *Phloeosinus tacubayae* was compared

Part of body		*Phloeosinus tacubayae*	*Phloeosinus canadiensis*	*Dendroctonus* ^b^	*Hylurgops* ^b^	*Scolytus* ^b^	*Conophthorus* ^b^	*Dryocoetes* ^b^	*Ips* ^b^	*Pityogenes*	*Pityophthorus*	*Bagous claudicans*	*Orobitis cyanea*	*Sciaphilus asperatus*
Head capsule	Dorsal	0	5	5	5	5	5	5	5	5	5	5	1	2
	Posterior	2	3	4	4	3	4-5	4	5	4	4-5	3	4	5
	Lateral	2	2	2	2	2	2	2	2	2	2	2	2	2
	Ventral	2	3	3	3	3	3	3	3	3	3	^a^	^a^	^a^
	Frontal	4	5	5	5	6	5	5	5	5	5	1	3	5
	Clypeal	2	2	2	2	2	2	2	2	2	2	2	1	2
	Labral	3	3	3	3	3	3	3	3	3	3	3	3	3
	Mandibular	2	2	2	2	2	2	2	2	2	2	2	2	1
	Head-free capsules	Retracted	Retracted	Free	Free	Retracted	Free	Free	Free	Free	Free	Free	Free	Free
	Epicranial sutures	Unmarked	Unmarked	Marked	Marked	Marked	Marked	Marked	Marked	Marked	Marked	Marked	Marked	Marked
	Pigmented head capsule	Less	Less	Major	Major	Less	Major	Major	Major	Major	Major	Major	Major	Major
	Sensillum chaetic in antenna	Long	Long	Short	Short	Long	Short	Short	Short	Short	Short	Short	Short	Short
Body	Ornamented body cuticle	Less	Less	In the last segments of the abdomen	longer and more visible setae	^a^	^a^	^a^	^a^	^a^	^a^	In the last segments of the abdomen	Less	Major
Prothorax	Pronotal	7	11	11	11	11	11	11	11	11	11-12	7	7	9
	Pleural	2	3	2	2	2	2	2	2	2	2	2	2	3
	Eusternal	2	3	3	3	3	4	3	4	3	3	1	1	2
	Mesosternal	1	1	1	1	1	1	1	1	1	1	^a^	^a^	1
	Pedal area	6	2	3-4	4	2	2	2	2	2	2	1	3	6
Meso- Metathorax	Prodorsal	2	1	1	1	1	1	1	1	1	1	1	1	1
	Postdorsal	1	5	5	5	5	5	5	5	5	5	3	1	4
	Alar area	^a^	2	2	2	2	3	2	3	3	3	1	^a^	1
	Spiracular area	2	1	1	1	1	1	1	1	1	1	2	2	2
	Spiracles	less excluded	less excluded	excluded	^a^	^a^	^a^	^a^	^a^	^a^	^a^	^a^	^a^	^a^
	Epipleural	1	1	1	1	1	1	1	1	1	1	1	1	2
	Pleural	2	1	1	1	1	1	1	1	1	1	1	1	2
	Eusternal	1	2	3	3	3	3	3	3	3	3	^a^	1	1
	Pedal area	0	3	3-4	4	2	2	2	2	2	2	1	3	5
Abd. segment I-VIII	Prodorsal I-VII	1	1	1	1	1	1	1	1	1	1	1	1	2
	Postdorsal I-VII	0	5	5	5	5	5	5	5	5	5	4	1	10
	Spiracular I-VII	1	2	2	2	2	2	2	2	2	2	1	1	2
	Mesosternal	2	2	2	2	2	2	2	2	2	3	3	1	2
	Epipleural I-VII	2	2	2	2	2	2	2	2	2	2	1	1	2
	Pleural VII-VIII	1	2	2	2	2	2	2	1	2	2	^a^	^a^	9
	Mesosternal VII-VIII	2	2	2	2	2	2	2	2	2	2	^a^	1	^a^
	Eusternal VII-VIII	2	1	1	1	1	1	1	2	1	1	2	2	2
Abd. segment IX	Anal area	*6*	2	2	2	2	2	2	2	2	2	2	^a^	3
	Tergal plate	Absent	Absent	Present	Absent	^a^	^a^	^a^	^a^	^a^	^a^	^a^	^a^	^a^

^a^Without enough information.

^b^The species does not appear because in the study from which the information was extracted, more than 1 species per genus was compared to determine the number of setae.

The patterns of morphology and chaetotaxy between *P. canadensis* and *P. tacubayae* described, suggest that *Phloeosinus* has unique attributes in the cephalic capsule that allow differentiating its larvae from those of other genera. For example, *Dendroctonu*s spp. Has unique transverse and rough areas in the cephalic capsule; *Hylurgops* members present a tubercle located in the middle part of the frons, and the other genera, such as *Conophtorus*, *Drycoetes*, *Ips*, *Pityogenes*, and *Pityophthorus*, also display common larval attributes among their members. Shared attributes within species of the same genera roughly suggest that these could constitute synapomorphies, as has been supported by phylogenetic studies in *Dendroctonus* (Víctor and Zúñiga 2014), however, further studies are needed to include a larger number of genera and species.

Larvae of *P. tacubayae* showed differences in chaetotaxy in some areas with respect to *P. canadensis* (see Table 2); *P. tacubayae* in the cephalic capsule has 2 posterior setae and *P. canadensis* 3, *P. tacubayae* has 3 setae on the maxillary stipes and 3 teeth in the mandible, whereas *P. canadensis* has 2 setae on the maxillary stipes and has only 2 teeth on the mandible, respectively. In the prothorax, the most marked differences in the number of setae were for *P. tacubayae* with 7 pronotal setae, while *P. canadensis* 11, in the pleural areal it has only 2 setae and *P. canadensis* 1; in the pedal area, *P. tacubayae* has 6 setae while *P. canadensis* 2; in the meso- and metathorax, the postdorsal area has 1 setae in *P. tacubayae* and 5 in *P. canadensis*; in the abdominal segments *P. tacubayae* has no postdorsal setae, meanwhile *P. canadensis* has 5. These morphological differences represent taxonomic characteristics to differentiate these species and suggest that *Phloeosinus* larval chaetotaxy is species-specific (Thomas 1957).

In this study, discrete characters were evaluated in the head, thorax, and abdomen of larvae and pupae, including body chaetotaxy, but no inter-instar variations were found. In beetle species such as those of the family Bruchidae there are differences among larval instars in body chaetotaxy (Li-Hsin Wu et al. 2012); which have been attributed to changes with food and shelter needs of instars causing the number of sensilla to vary (Pfaffenberger 1985). Bark beetles have evolved to live and develop their complete life cycle within plant tissues, which has caused their larvae to reduce their appendages, becoming apodous and having fewer cuticular sensillae (Wood 2007). Unfortunately, there are no studies that compare the differences in chaetotaxy of the different larval instars in bark beetles, we hypothesize that the absence of changes in the sensilla among larval instars of *P. tacubayae* is due that they do not present habitat and nutritional changes throughout their development. The only particular morphological difference that occurred in the third instar is the apical extension of abdominal segments before the larva initiates the metamorphosis.

### Larval Antenna

One of the most outstanding aspects of the description of the chaetotaxy in this study was the inclusion of the antennal sensilla, these structures have been widely studied in adults of Scolytinae (Payne et al. 1973, Dickens et al. 1977, Whitehead 1981, López et al. 2014). Sensilla are the basic units of their sensory system and thanks to them the bark beetles have a gustatory response for the selection of food and recognition of conspecifics (Zacharuk 1980, Ranger et al. 2017). In *P. tacubayae* the antenna of the larva is formed by a sensorium (*se*), 3 basiconica sensilla (*sb*), a long chaetica sensilla (*sc*) (Figs. 7C and 8B). According to our observations, both *se* and *sb*, in *P. tacubayae* present a multiporous wall, a flat tip, a smooth surface, and a socket base, characteristics that are associated with the perception of chemical stimuli (Dickens and Payne 1978). The location of conical sensorium (*se*) and (*sb*) in this study resembles that of sensilla basiconica (B.a.1) and the sensilla twig basiconica I and II (T.b.1, T.b.2) in *Ips typographus* (Linnaeus, 1758) and *Ips subelongatus* (Motschulsky, 1860) (Xia Shi et al. 2020), which also were associated with chemical functions, to detection of toxic compounds and response-promoting chemicals from their food, while the T.b.1 and T.b.2 sensilla are olfactory receptors.

The longest sensillum in the antenna of *P. tacubayae* (*sc*) presents aporous wall, sharp-tipped, smooth wall, and lacy base, characteristics associated with mechanoreceptive functions (Payne et al. 1973, Reinhard 2006, Xia Shi et al. 2020). These sensilla have not been reported in the antenna of Scolytinae larvae; in the most detailed description of antennal sensillum of larvae corresponding to *Ips* species, this sensillum is not present (Xia Shi et al. 2020). The location of *sc* also resembles that of chaetica sensillum from the antenna of *Acanthoscelides obtectus* (Say) (Coleoptera: Bruchidae) (Pfaffenberger 1985), whose function is associated with the detection of vibrations of neighboring larval chambers. Its presence in *P. tacubayae*, could be also related to a similar function, to promote the larval feed in free tunnels and not cross each other, and thus avoid intraspecific competition and cannibalism (Kirkendall 1983, Mendel 1984). Finally, the microtrichia is another important element in the antenna that serves to protect and keep clean the surface of important sensory receptors (Pfaffenberger 1985); in *P. tacubayae* the microtrichia seem to have this function since they cover the surface of the sensilla chaetica and the sensorium.

### Pupae

It is the least studied stage in Scolytinae, the only source for comparative purposes corresponds to the work of Hopkins (1909). Based on that study it was possible to recognize notable differences between *D. valens* and *P. tacubayae*. The pupa of the second species presented a lower number of setiform tubercles compared to *D. valens* (Hopkins 1909), and both species display a lower number of these elements when compared to other Curcuilonidae subfamilies as Lixinae (Stejskal et al. 2014), Entiminae (Gosik et al. 2019b), Curculioninae (Gosik et al. 2017); these subfamilies present quadruple the number of setae all over the body compared with *P. tacubayae*. This reduction in setae number may be attributed to body size and morphological adaptations to sense changes in the environment or predators as well as to food and specialization of the life cycle. For example, the pupae of curculionid species that develop their life cycle in plant organs and tissues, more exposed to desiccation, present a greater number of setae on the body such as *Larinus vulpes* (A. G. Olivier, 1807 (Coleoptera: Curculionidae) (Skuhrovec et al. 2017), while endophytic species that develop their life cycle inside plant tissues and organs have a lower number of setae or these are reduced in size, such as *Eurymmatus *mariae** (Roger, 1856) (Coleoptera: Curculionidae), which is a rare species of saprophytic weevil of *Pinus* (L, 1753) (Pinales:Pinaceae) genus trees. This species pupates inside the wood, where the insects have greater protection from external agents such as abrupt changes in climate (Skuhrovec et al. 2017, Gosik and Wanat 2021).

### Sexual Dimorphism

In most members of the genus *Phloeosinus*, the adults show marked sexual dimorphism in the sculptural elements of frons, interstriae I, and III of the elytral declivities (Wood 2007). In *P. tacubayae* some attributes of the head and elytral declivity have been defined for this purpose, for example, proportions and sculpture of the frons, shape, vestiture, and pubescences of the elytral declivity (Hopkins 1905, Blackman 1942, Wood 1982). However, our morphological comparisons of adult males and females of this species did not allow us to recognize conspicuous differences between the sexes in these body regions. According to our results, a reliable way to sex specimens of this species is by observation of the last abdominal tergites. In females, the seventh tergite completely covers the eighth tergite, while in males, the seventh tergite is shorter than in females and the eighth tergite is completely visible. The last abdominal tergites have been useful in recognizing sexes in other beetle species (Wood 1982, Riddick and Schaefer 2005). In *Dendroctonus* the seventh abdominal tergite has been used as a morphological character to differentiate sexes (Lyon 1958, Mendoza-Correa and Zúñiga 1991, Rios-Reyes et al. 2009). This highlights the importance of identifying morphological characters based not only on the most visible external part of the body but also on other structures below the elytra that can provide valuable information.

### Time of Development

*Phloeosinus tacubayae* develops its life cycle in Mexican Cypress trees, under conditions of contamination and dehydration (Cervantes-Espinoza et al. 2022), in sites with high tree density, these species promote high tree mortality (Cibrián-Tovar et al. 2000). Knowledge of the time of development is crucial for planning control measures, as this would disrupt the initial stages. In our observations, we studied the development of immature stages and the time needed to complete different biological processes in the laboratory (hatching, pupation, metamorphosis, maturation, and emergence), by exposing them to typical conditions (2,300 masl;15–25 °C). The time required for full development from egg to adult stage of *P. tacubayae* was 40 days; a similar period to that of *P. armatus* and *Phloeosinus aubei* (Perris, 1855), which is about 49 days when the first adults begin to emerge (Kirkendall 1983, Baruch et al. 2017). However, the insect can be within the tree bark for up to 120 days during the emergence period, which should be considered in management strategies. Other species such as *P. aubei* (BelHabib et al. 2009), *Dendroctonus micans* (Kugelann, 1794) (Lévieux et al. 1985) also took 41 days to form gallery systems and complete their life cycle, but adult emergence time is not reported.

Larval development in *Phloeosinus tacubayae* was faster than pupae, around 15 days, and the pupae take the longest time to develop (>20 days). These data will serve as a model to test under different temperatures and evaluate how environmental variation impacts the development time of the different instars.
